# Editorial: Telomere Flexibility and Versatility: A Role of Telomeres in Adaptive Potential

**DOI:** 10.3389/fgene.2021.771938

**Published:** 2021-10-04

**Authors:** Radmila Čapková Frydrychová, James M. Mason, Vratislav Peska

**Affiliations:** ^1^ Institute of Entomology, Biology Centre of the Czech Academy of Sciences, České Budějovice, Czechia; ^2^ Faculty of Science, University of South Bohemia, České Budějovice, Czechia; ^3^ Retired, Greensboro, NC, United States; ^4^ Department of Cell Biology and Radiobiology, Institute of Biophysics of the Czech Academy of Sciences, Brno, Czechia

**Keywords:** telomere, telomerase, stress response, subtelomere, alternative pathway

Telomeres are key structures for chromosome end capping and concurrently one of the key factors that guard the stability and integrity of the whole genome (Blackburn 1990). Telomere function seems to be universal and essential for all eukaryotes, and its dysfunction is associated with a wide range of detrimental cellular and physiological consequences (Blackburn 1991). The fact that telomere DNA sequence is highly conserved for large taxonomic groups and that telomerase is the prime mechanism for telomere maintenance in most tested eukaryotes (Gomes et al., 2010) might evoke a more or less unified and rigid concept of telomere structure and function. However, more detailed observations reveals that telomere structure and function can be very dynamic and flexible, as documented by frequent evolutionary novelties in plant telomere motifs or telomerase-independent telomere maintenance pathways in numerous insects (Mason et al., 2011; Mason et al., 2016; Fajkus et al., 2019; Peska and Garcia 2020; Fajkus et al., 2021). Further, changes in the composition of telomere sequences or telomere maintenance activity are found as a part of telomere adaptive response to environmental or physiological stress exposure (Barry et al., 2003; Korandová et al., 2018). Even more interestingly, it is proposed that telomere dysfunction, which leads to loss of telomere capping function and genomic instability, might initiate the speciation process through induced genomic rearrangements (Stindl 2004; Stindl 2014; Prušáková et al., 2021).

The adaptive function of telomeres has been widely studied in pathogenic microorganisms such as *Trypanosoma* sp., in which DNA recombination-mediated antigenic variation and its regulation by telomeres were shown, and telomere components were suggested as potential therapeutic targets for treating pathogen infections (Li et al., 2012; Jehi et al., 2014). These suggestions are related to the fact that subtelomeres commonly harbor genes with high adaptive potential to sustain the pathogen within host organisms (Barry et al., 2003); diversification of these genes is believed to act through enhanced chromosomal rearrangements in subtelomere regions (Robinson et al., 1999; Barry et al., 2003; Keely et al., 2005). Subtelomeres are generally considered to be highly dynamic, and it has long been proposed that compromised telomere function, which is elicited by certain environmental stresses, might trigger chromosomal polymorphisms at subtelomere regions resulting in rapid adaptation of the organism to a novel environment (McEachern 2008; Mason and McEachern 2018).

New evidence of “adaptive telomere failure” is framed by an article in our series entitled “*Telomere flexibility and versatility: a role of telomeres in adaptive potential*” by Rahnama et al. The authors focus on telomere instability in the blast fungus, *Pyricularia oryzae*. Following previous studies (Starnes et al. 2012; Rahnama et al., 2020), they showed that telomere instability in the species is driven by retrotransposon insertion into telomere repeats, which generates interstitial telomere sequences that potentiate frequent chromosomal rearrangements involving telomeres, as well as exchanges with the internal genome regions. These rearrangements may have adaptive advantages. Importantly, based on TERT knockout experiments, the authors document that sequence polymorphism in the region is induced when telomere functioning is threatened.

In contrast to many other fungi, telomere sequences in budding and fission yeasts not only differ from the canonical TTAGGG repeat but also frequently vary (Shampey et al., 1984; Peska et al., 2021; Červenák et al., 2021). Although the underlying reason for the sequence variation in the yeasts is unclear, it documents the capacity of fungal telomeres to adapt to the sequence alterations. The protein composition of fungal telomeres displays a high rate of structural and functional divergence, and significant changes in the protein composition are seen even in fungi with the canonical 6-bp telomere unit, as discussed by Lue in his review article. Using the example of the Tay1 family proteins, the author presents a scenario of functional diversification for developing a new telomere protein or a protein with a new telomere function. This model also shows how telomere proteins might be available to evolve new protein-protein or nucleic acid-protein interactions and act as a key factor allowing telomere flexibility and adaptability.

Due to their G-rich content, telomeres are exceptionally prone to oxidative stress-induced damage, principally causing replication fork arrest and leading to telomere dysfunction (Coluzzi et al., 2019). It had been shown that chronic oxidative stress and persistent telomeric double-strand breaks (DSBs) activate the recombination-dependent alternative lengthening of telomeres (ALT) (Coluzzi et al., 2017; Liu et al., 2018), although telomere-specific DSBs are considered irreparable (Fumagalli et al., 2012; Hewitt et al., 2012). In our article collection, Nelson et al. demonstrate that although telomeric DSBs induced during the G1cell cycle stage in human cells lack evidence of classic non-homologous end-joining or homologous recombination (HR)-dependent repair, they lead to the formation of extensive tracks of 5’ C-rich single stranded telomeric DNA, which have been reported as a marker of the ALT pathway. As shown in this study, the resected broken ends are bound by the complementary telomeric RNA, TERRA, which likely protects the ends until telomerase-mediated or HR-dependent telomere elongation are activated during the S/G2 cell cycle stage.

Finally, a review article by Sellami et al. discusses the impact of various forms of physical activity and exercise on telomere maintenance and global epigenetic modifications and their role in anti-aging strategies in humans. The main conclusion of the article is that in contrast to over-loaded training, regular and moderate physical activity increases the activity of telomerase and attenuates telomere shortening, and aerobic exercise training appears the most effective in conserving telomere length when compared to less aerobic activities.

Collectively, telomeres might change and embrace new features to maintain their integrity and functionality, guard cell viability, or even provide an adaptive advantage to the whole organism when exposed to unfavorable environmental or physiological conditions. Realizing all this telomere flexibility and versatility, it is tempting to speculate about the complexity of telomere biology and search for further biological relevance and consequences of the whole phenomena.
